# Genome-wide identification and expression analyses of the pectate lyase (*PEL*) gene family in cotton (*Gossypium hirsutum L*.)

**DOI:** 10.1186/s12864-018-5047-5

**Published:** 2018-09-10

**Authors:** Huiru Sun, Pengbo Hao, Qiang Ma, Meng Zhang, Yuan Qin, Hengling Wei, Junji Su, Hantao Wang, Lijiao Gu, Nuohan Wang, Guoyuan Liu, Shuxun Yu

**Affiliations:** 1 College of Agronomy, Northwest A&F University, Yangling, 712100 China; 2 State Key Laboratory of Cotton Biology, Institute of Cotton Research of CAAS, Anyang, 455000 China

**Keywords:** *Gossypium* spp., Pectate lyase (PEL), Gene expression patterns, Pollen development, Fiber, Auxin

## Abstract

**Background:**

Pectin is a major component and structural polysaccharide of the primary cell walls and middle lamella of higher plants. Pectate lyase (PEL, EC 4.2.2.2), a cell wall modification enzyme, degrades de-esterified pectin for cell wall loosening, remodeling and rearrangement. Nevertheless, there have been few studies on *PEL* genes and no comprehensive analysis of the *PEL* gene family in cotton.

**Results:**

We identified 53, 42 and 83 putative PEL genes in *Gossypium raimondii* (D5), *Gossypium arboreum* (A2), and *Gossypium hirsutum* (AD1), respectively. These PEL genes were classified into five subfamilies (I-V). Members from the same subfamilies showed relatively conserved gene structures, motifs and protein domains. An analysis of gene chromosomal locations and gene duplication revealed that segmental duplication likely contributed to the expansion of the *GhPELs*. The 2000 bp upstream sequences of all the *GhPELs* contained auxin response elements. A transcriptomic data analysis showed that 62 *GhPELs* were expressed in various tissues. Notably, most (29/32) *GhPELs* of subfamily IV were preferentially expressed in the stamen, and five *GhPELs* of subfamily V were prominently expressed at the fiber elongation stage. In addition, qRT-PCR analysis revealed the expression characteristics of 24 *GhPELs* in four pollen developmental stages and significantly different expression of some *GhPELs* between long- and short-fiber cultivars. Moreover, some members were responsive to IAA treatment. The results indicate that *GhPELs* play significant and functionally diverse roles in the development of different tissues.

**Conclusions:**

In this study, we comprehensively analyzed *PEL*s in *G. hirsutum*, providing a foundation to better understand the functions of *GhPELs* in different tissues and pathways, especially in pollen, fiber and the auxin signaling pathway.

**Electronic supplementary material:**

The online version of this article (10.1186/s12864-018-5047-5) contains supplementary material, which is available to authorized users.

## Background

Plant growth and development along with cell expansion and division give rise to the morphogenesis of many organs, tissues and cells [1]. The cell wall is a significant structure that maintains the cell’s internal pressure, stability, tensile strength and defense [2]. Pectin, which is mainly present in the primary cell wall and middle lamella, is a polysaccharide containing a linear backbone of α-1,4-linked galacturonic acid residues that forms a matrix embedded in cellulose and hemicellulose [3, 4]. Homogalacturonan (HG), a major component of pectin, can be degraded by various pectinases, including the polygalacturonases (PGs), pectin acetylesterases (PAEs), pectin methylesterases (PMEs) and pectate lyases (PELs) [5]. PEL, (EC 4.2.2.2) depolymerizes HG through a β-elimination reaction, generating 4,5-unsaturated oligosacceharides [6, 7].

PELs have been widely identified in plant pathogenic bacteria, such as *Erwinia chrysanthemi,* which causes soft-rot disease in many plants [8]. In many higher plants, expression of *PEL-like* genes has also been found in a variety of tissues, including the pollen of tomato, tobacco and Japanese cedar [9–11], ripening fruits of strawberry and banana [6, 12–14], tracheary elements of Zinnia [15], fiber of cotton [16], xylem of poplar [17] and lateral roots of Arabidopsis [18].

In Arabidopsis, a genome-wide analysis and an analysis of promoters showed that PEL-like genes (*AtPLLs*) play an important role in the development of flowers and cell separation [19, 20]. In pollen development, *PELs* may regulate the loosening and degradation of the pollen cell wall [11]. In the process of fruit ripening, *PELs* modify the pectin structure in the cell wall [13]. In *Populus*, most of the 30 *PtPLs* are highly expressed in xylem, performing important functions during the development of wood [17]. In Arabidopsis, the increased expression of *AtPLAs* (*PEL* genes) promotes the degradation of the pectin-rich middle lamella during lateral root emergence [18]. In rice, the *DEL1* gene (a *PEL* gene, LOC_Os10 g31910) regulates plant growth and leaf senescence through controlling cell numbers and triggering reactive oxygen species accumulation [1]. Auxin, an important plant hormone, regulates plant growth and development by advancing acid-mediated changes in the cell wall [21, 22]. The acidification of the cell wall activates the expansins and PMEs, which causes loosening of the cell wall [2]. Many studies have revealed that *PELs* respond to IAA treatment [13, 18, 19, 23, 24]. These studies indicate that *PEL* genes exhibit extensive functions in plant growth and development and participate in the auxin regulation pathway.

Cotton is the most important natural fiber crop. Pectins are responsible for 25% of the cell wall components of rapidly elongating cotton fibers (8 days postanthesis (DPA)), indicating that the structure and configuration of pectin can influence fiber quality [25, 26]. A study examining *GhPEL* indicated that this gene is crucial for the normal elongation of cotton fibers through degradation of de-esterified pectin, facilitating the loosening of the cell wall [16].

However, there have been few studies related to *PEL* genes in plants, and they have mainly focused on the functional analysis of individual genes. In cotton, most of the *PEL* genes are unknown. At present, there are no available genome-wide analyses of the cotton *PEL* gene family. With the completion of the genome sequencing of *G. raimondii*, *G. arboreum* and allotetraploid cultivated cotton (*G. hirsutum* cv TM-l), we can now perform a comprehensive analysis of *PELs* in cotton [27–31]. In this study, we predicted the *PELs* of three *Gossypium* species and analyzed their gene structure, phylogenetic tree, expression characteristics and other features. The results provide a reference for the potential functions of *PELs* in plant growth and development.

## Results

### Genome-wide identification of PELs in *G. raimondii, G. arboreum and G. hirsutum*

Based on the conserved Pec_lyase_C (Pfam00544) domain and SMART analyses, we identified 53, 42, and 83 full-length putative PELs in *G. raimondii, G. arboreum* and *G. hirsutum* TM-1, respectively. According to their locations on the chromosomes, the family members of the three species were designated *GrPEL1* to *GrPEL53*; *GaPEL1* to *GaPEL42*; and *GhPEL1* to *GhPEL83*, respectively. The lengths of the putative GhPEL proteins varied from 171 (GhPEL20_At) to 680 (GhPEL52_Dt) amino acids (aa), while those of GaPELs ranged from 136 aa (GaPEL36) to 680 aa (GaPEL14), and those GrPELs varied from 222 aa (GrPEL36) to 511 aa (GrPEL27). The predicted Mw, pI, GRAVY and subcellular localization of the protein sequences are shown in Additional file 1: Table S1.

### Phylogenetic analysis of the *PEL* gene family

**Fig. 1:**
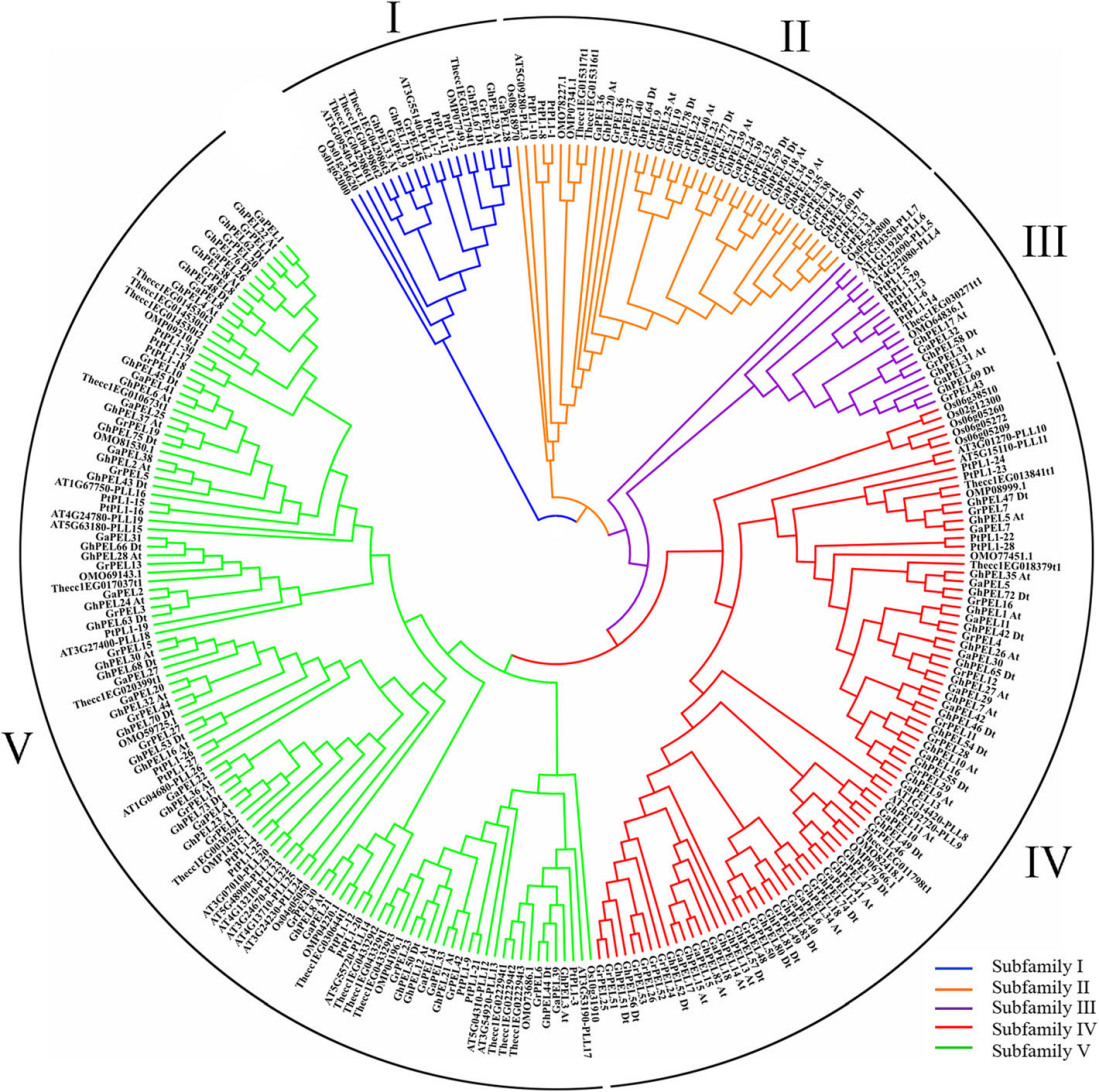
Phylogenetic tree of PEL proteins. The 285 predicted protein sequences from *Gossypium raimondii*, *G. arboreum*, *G. hirsutum*, *Arabidopsis*, *Corchorus olitorius*, *Theobroma cacao*, *Oryza sativa* and *Populus* were aligned with ClustalX 2.0, and the phylogenetic tree was generated using MEGA 6.0 via the neighbor-joining (NJ) method with 1,000 bootstrap replicates. Five subfamilies of PELs are indicated using different line colors

To examine the evolutionary relationships of the PEL proteins and classify them into subfamilies according to the established subfamilies in Arabidopsis, we performed a phylogenetic analysis of 285 PEL protein sequences from *G. raimondii* (53), *G. arboreum* (42), *G. hirsutum* (83), *Arabidopsis thaliana* (26), *Corchorus olitorius* (16), *Theobroma cacao* (24), *Oryza sativa* (11) and *Populus trichocarpa* (30) to construct an unrooted phylogenetic tree. The PEL proteins were classified into 5 subfamilies (I, II, III, IV and V) (Fig. 1 and Additional file 2: Table S2). Subfamily IV and subfamily V were the two largest subfamilies and contained 101 and 103 PEL members, respectively, while both subfamily I and subfamily III contained only 20 PEL members. The PEL proteins of *Oryza sativa*, a monocot, were distant from the PEL proteins of the other dicot plants. These results indicated that *PELs* might have evolved in different directions and expanded to exhibit diverse functions among various species.

### Distribution and gene duplication events of *PELs*

**Fig. 2:**
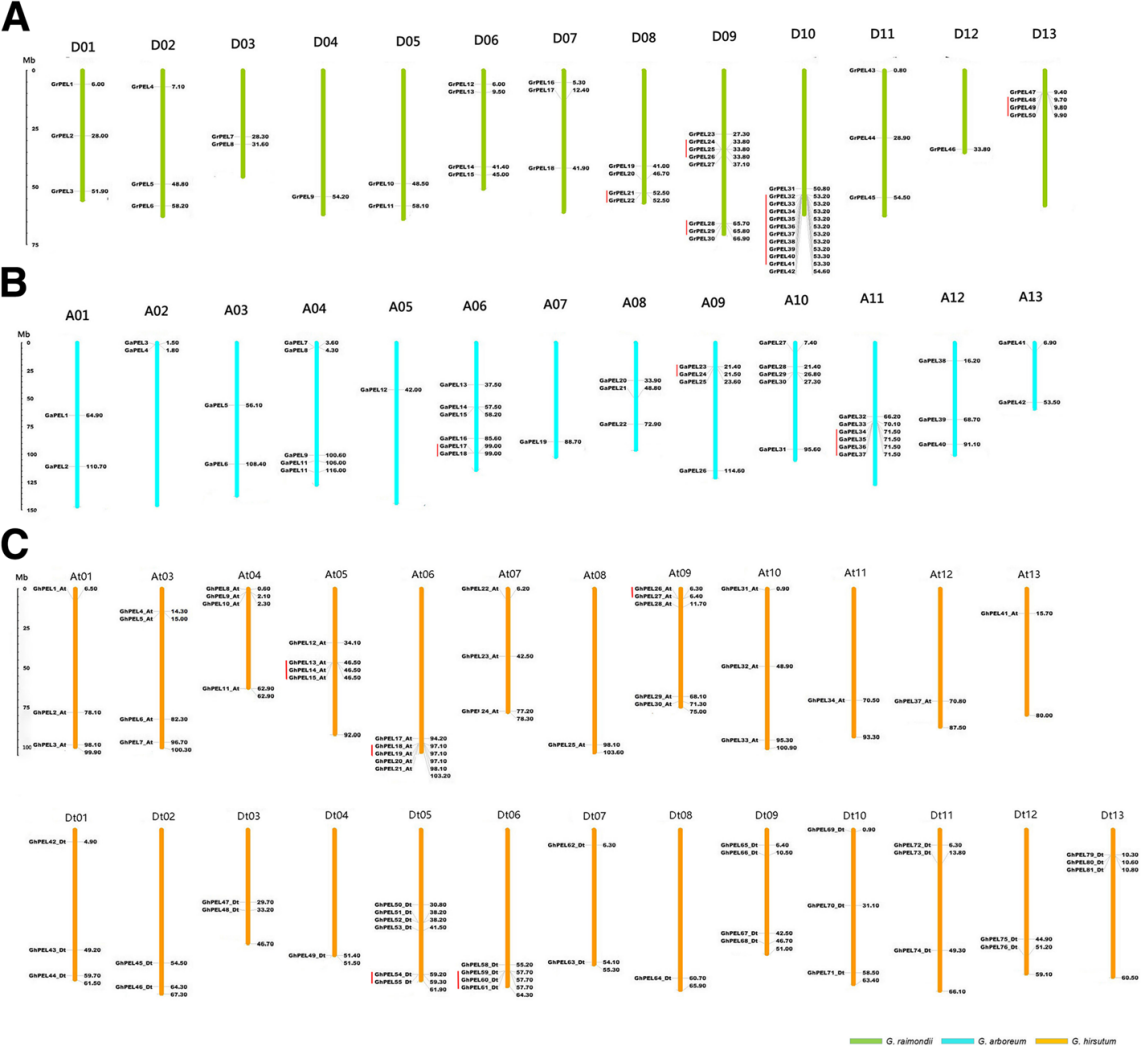
Chromosomal distribution of *PELs* from *Gossypium raimondii* (**a**), *G. arboreum* (**b**), and *G. hirsutum* (**c**). The scale represents megabases (Mb). The chromosome numbers are indicated above each vertical bar. The putative *PELs* are indicated on the different chromosomes. Green, blue and yellow bars represent the physical maps of *Gossypium raimondii* (**a**), *G. arboreum* (**b**), *G. hirsutum* (**c**), respectively. Red lines show the gene pairs involved in tandem duplication

The chromosomal distributions of *GrPELs, GaPELs* and *GhPELs* were determined according to their genomic locations (Additional file 3: Table S3). In *G. raimondii*, 50 *GrPELs* were unevenly anchored on 13 chromosomes, while 3 genes (*GrPEL51-GrPEL53*) were located on scaffolds. D10 contained the most *GrPELs* (12), followed by D09 with 8 *GrPELs*. However, there was only one *GrPEL* on D04 and D12 (Fig. 2a). In *G. arboreum*, 42 *GaPELs* were located on 13 chromosomes. Both A06 and A11 contained the most *GaPELs* (6 each). In contrast, both A05 and A07 only contained one *GaPEL* (Fig. 2b). A total of 71 *GhPELs* were mapped to the 25 *G. hirsutum* chromosomes, with the exception of At02, while 12 other *GhPELs* were located on unassembled scaffolds. The distribution of *GhPELs* on each chromosome was highly uneven: D05 contained the most *GhPELs* (7); At06 and At09 contained 5 *GhPELs*; and the other 22 chromosomes contained one to four *GhPELs* (Fig. 2c).

**Fig. 3:**
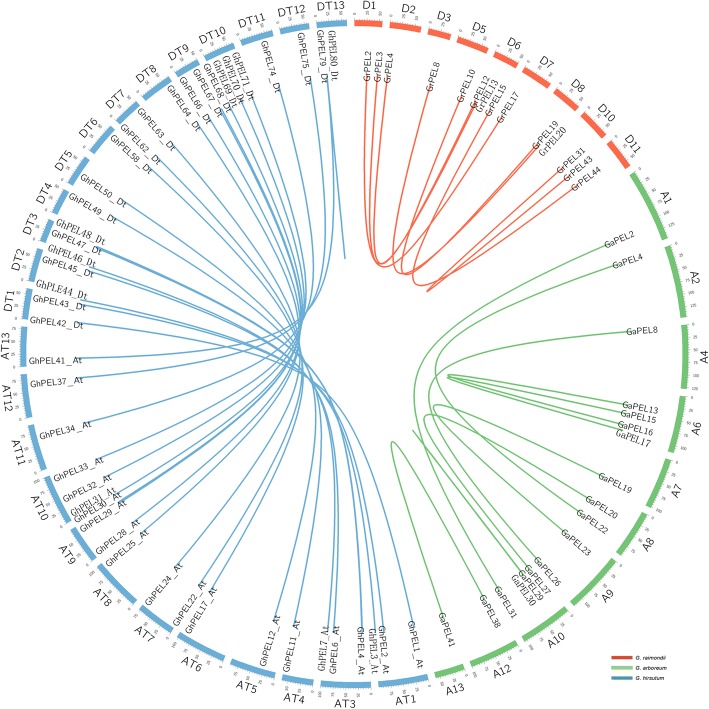
Circos figure of gene pairs of segment duplication in *GrPELs*, *GaPELs* and *GhPELs*. The chromosomes of *Gossypium raimondii*, *G. arboreum* and *G. hirsutum* are filled with red, green, and blue, respectively. Gene pairs involved in segment duplication are linked by a line

Previous studies have indicated that gene duplication events are vital to gene family expansion and occur along with plant genome evolution [32]. In the present study, a gene duplication analysis was performed to investigate the expansion mechanism of the *PEL* gene family in the three *Gossypium* species. In general, gene duplication events include tandem and segmental duplications. A total of 14, 13 and 35 gene duplication pairs were identified in *G. raimondii*, *G. arboreum* and *G. hirsutum*, respectively, accounting for 71.7%, 66,7% and 86.7% of the *PEL* gene family (Additional file 4: Table S4). Based on sequence similarity and chromosomal location, 5, 3 and 5 gene duplication pairs were determined to represent tandem duplication events in *G. raimondii*, *G. arboreum* and *G. hirsutum,* respectively, while 49 other pairs represented segmental duplication events; these pairs are shown in Fig. 3, except for the gene pairs located on unassembled scaffolds. A total of 13 clusters of tandem duplication were located on D08 (*GrPEL21*/*GrPEL22*), D10 (*GrPEL32*-*GrPEL41*), D13 (*GrPEL48*-*GrPEL50*), D09 (*GrPEL24*-*GrPEL26* and *GrPEL28*/*GrPEL29*), A06 (*GaPEL17*/*GaPEL18*), A09 (*GaPEL23*/*GaPEL24*), A11 (*GaPEL34*-*GaPEL37*), At05 (*GhPEL13_At*-*GhPEL15_At*), At06 (*GhPEL18_At*/*GhPEL19_At*), At09 (*GhPEL26_At*/*GhPEL27_At*), Dt05 (*GhPEL54_Dt*/*GhPEL55_Dt*) and Dt06 (*GhPEL59_Dt*-*GhPEL61_Dt*) (Fig. 2). These results indicated that gene duplication, especially segmental duplication, played an irreplaceable role in the expansion of the *PEL* gene family in the three *Gossypium* species.

To investigate the selection pressure for the segmental duplication of *PEL* gene pairs, the Ka/Ks ratio was calculated. The results showed that the Ka/Ks ratios for most of the segmental duplications of *PEL* gene pairs were less than 1.0, indicating that they had experienced purifying selection pressure after gene duplication events (Additional file 5: Table S5). Because of the constraints of purifying selection on divergence, most of the segmental duplications of the *PEL* pairs might exhibit similar functions. Only *GhPEL2_At*/*GhPEL43_Dt* presented a Ka/Ks ratio greater than 1, demonstrating that this *GhPEL* pair had undergone positive selection during cotton evolution.

In addition, divergence time analysis was performed between the segmentally duplicated *PEL* pairs (Additional file 5: Table S5). In *G. raimondii* and *G. arboreum*, the timing of the occurrence of the segmental duplication of *PEL* pairs was inferred to be 8.48–171.46 million years ago (MYA), with an average of 91.73 MYA, and 18.35–145.46 MYA, with an average of 85.73 MYA, respectively. In *G. hirsutum*, the timing of the occurrence of the segmental duplication *PEL* pairs was presumed to be 1.00–32.75 MYA, with an average of 10.24 MYA.

### Conserved domains and amino acid sites of GhPELs

The Pec_lyase_C domains and signal peptides of the PEL sequences were investigated and shown according to the phylogenetic tree of the GhPELs (Additional file 6: Figure S1). All of the GhPELs contained a Pec_lyase_C domain, indicating that this domain was conserved. Four Asp residues, one Cys residue, one Arg residue, and five additional amino acid residues (Asp, His, Thr, Pro and Arg) in the Pec_lyase_C domain involved in Ca^2+^-binding, disulfide bonds, catalysis and substrate binding, respectively, were found to be highly conserved, indicating that these amino acid sites were significant for the function of *GhPELs* (Additional file 7: Figure S2). Most members of the GhPELs (77.1%) exhibited the predicted signal peptide. However, the members of subfamily I exhibited no signal peptide, which was consistent with a previous study on PtPLs [17].

### Gene structure and conserved protein motifs of GhPELs

**Fig. 4:**
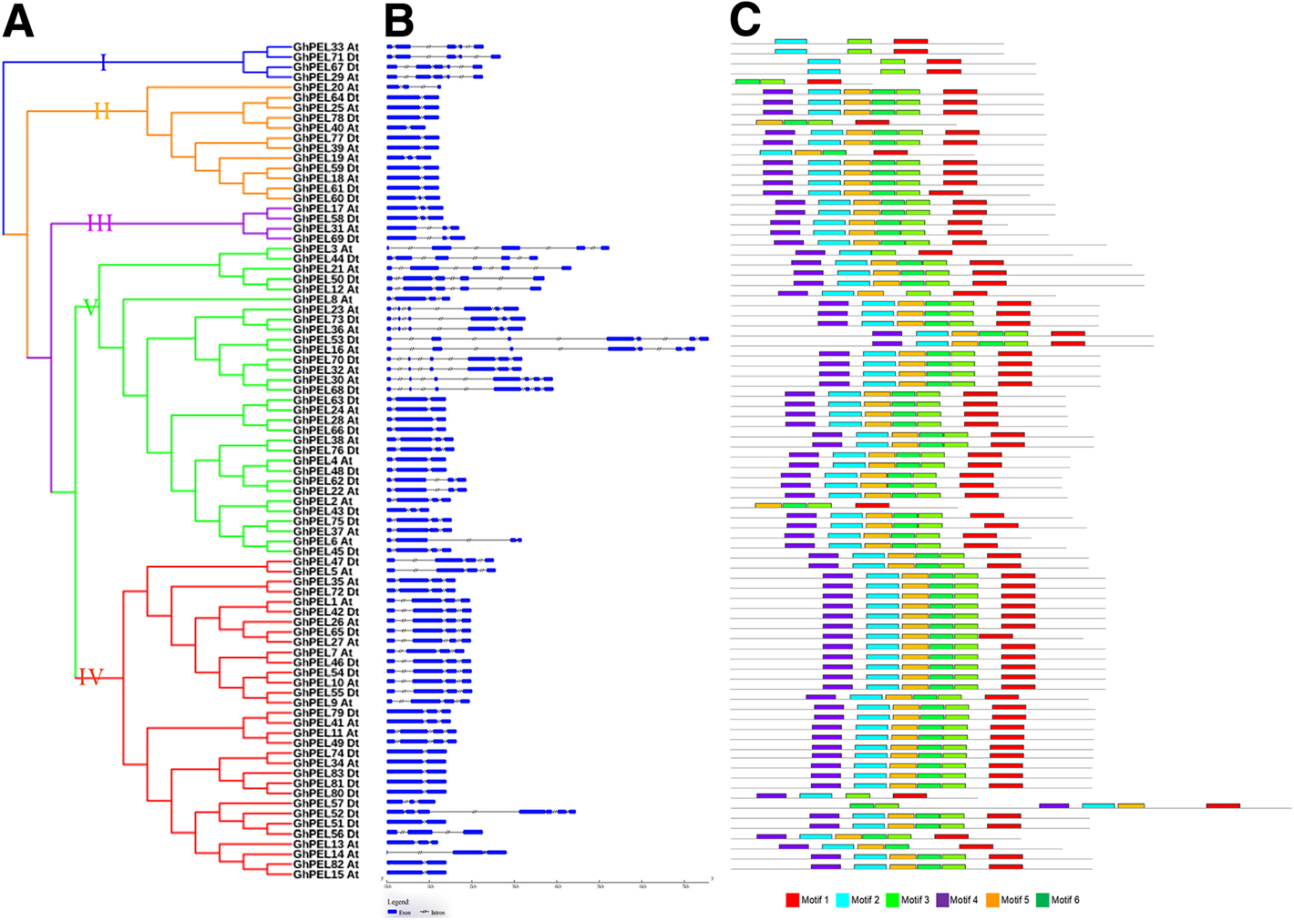
Phylogenetic relationships, gene structure and motif analysis of *GhPELs*. **a** Phylogenetic analysis of GhPEL proteins using MEGA 6.0 via the neighbor-joining (NJ) method with 1,000 bootstrap replicates. **b** Exon-intron organization of the GhPEL family. The exons and introns are indicated with blue filled boxes and black lines, respectively. **c** Six motifs of GhPEL proteins determined using MEME. Different colors represent different motifs

To further understand the conservation and diversification of the *GhPELs*, their exon-intron structures and conserved motifs were investigated and were shown in Fig. 4. The members of subfamily I and subfamily III each contained 5 exons and 3 exons, respectively. Most members of subfamily II exhibited 2 exons, except for *GhPEL20_At*, *GhPEL19_At* and *GhPEL60_Dt*, which presented 3 exons. More than half (17/32) of subfamily IV contained 4 exons, while the other members displayed 2 or 3 exons, with the exception of *GhPEL52_Dt*, with 7 exons. The exon numbers of subfamily V members varied from 3 to 7. The exon numbers were highly diverse among *GhPELs* (ranging from two to seven), indicating that functions of *GhPELs* might be diverse. However, closely related *GhPELs* showed similar exon-intron structures, and these genes might play similar roles in plant growth and development (Fig. 4b).

We identified 6 conserved motifs of the GhPEL proteins using MEME (Fig. 4c). Within a given subfamily, most of the members exhibited similar motif construction. Motifs 1, 5, 3 and 6 within the conserved Pec_lyase_C domain were identified in most of the GhPEL proteins. A total of 90.4% of the GhPEL proteins (except for the members of subfamily I and GhPEL20_At, GhPEL40_At, GhPEL19_At, and GhPEL43_Dt) contained motif 4. Motif 2 existed in 96.4% of the GhPEL proteins (except for GhPEL20_At, GhPEL40_At, and GhPEL43_Dt). Taken together, the motifs and their arrangement showed a high conservation in the GhPEL family.

### Analysis of cis-elements related to auxin in putative *GhPEL* promoter regions

Many studies have shown that *PELs* respond to auxin treatment. Therefore, the 2000 bp upstream regions from the initiation codons (ATG) of *GhPELs* were scanned in the PLACE database to obtain the cis-acting elements related to auxin. The results showed that all of the putative *GhPEL* promoter regions contained at least one of the six major auxin-responsive cis-elements: S000024, S000026, S000270, S000273, S000360, and S000370. In addition, the putative promoter region of *GhPEL63_Dt* contained the largest number (15) with all six auxin-responsive cis-elements. Ca^2+^- mediated crosslinking of demethyl-esterified HG, the substrate of PEL, can change cell wall structure, which is important for cell expansion and division [33]. A Ca^2+^-responsive cis-element (S000501) and calmodulin-binding/CGCG box (S000507) were identified in 35 (42.2%) of the putative promoter regions of *GhPELs* (Additional file 8: Table S6). This result indicated that some *GhPELs* might alter the configuration of the cell wall with Ca^2+^.

### Expression patterns of *GhPELs* in different tissues

**Fig. 5:**
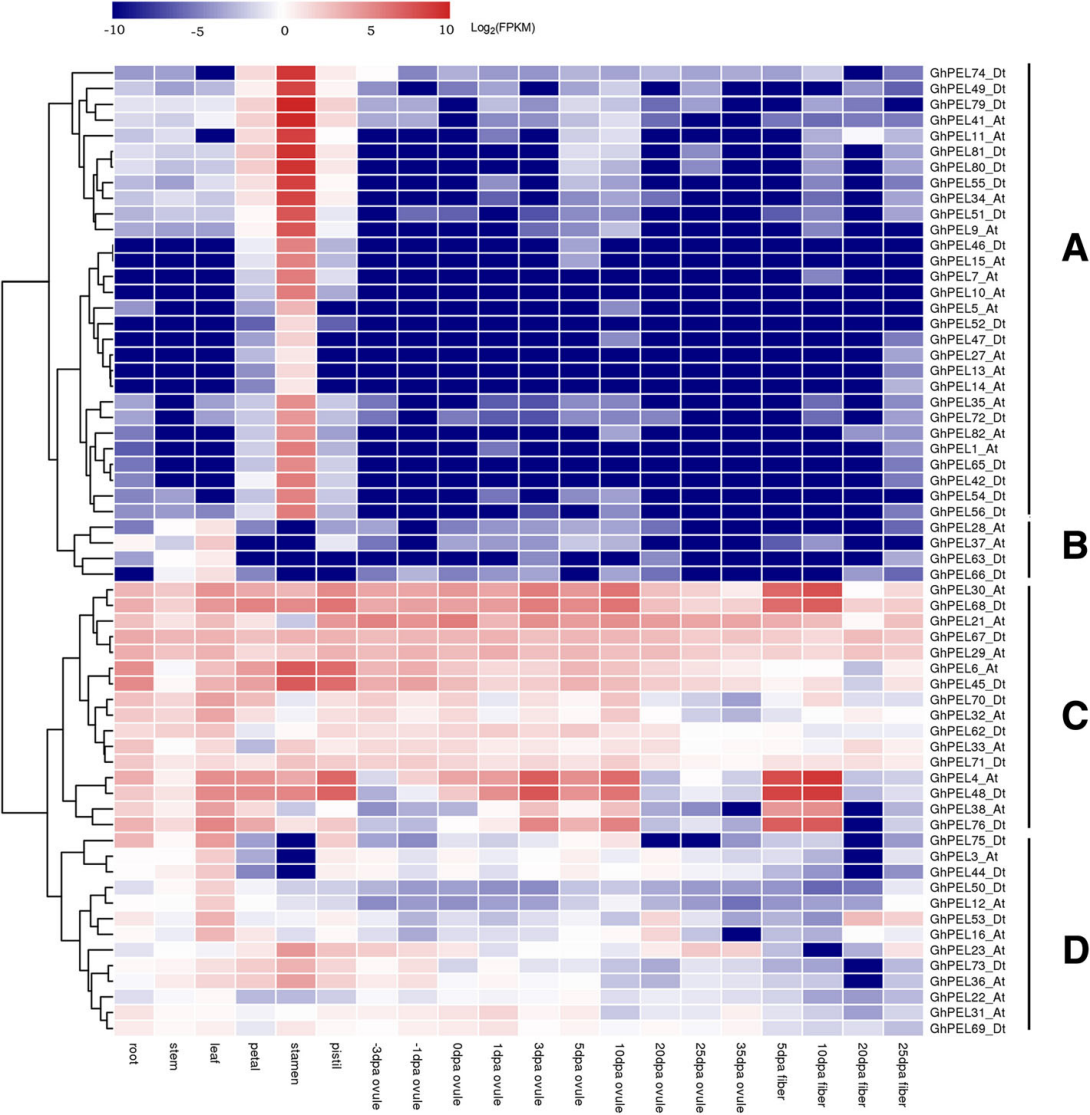
Expression profiles of *GhPELs* in different tissues. The tissues are shown on the bottom; the genes are shown on the right; and the phylogenetic relationships are shown on the left and have been classified into four groups (**a**-**d**). Scale bars at the top represent log_2_-transformed FPKM values. -3 DPA to 35 DPA indicates − 3, − 1, 0, 1, 3, 5, 10, 20, 25 and 35 days after anthesis

To explore the possible biological functions, the spatio-temporal expression patterns of *GhPELs* were investigated in different tissues, including root, stem, leaf, petal, stamen, pistil, ovules and fibers at various developmental stages. Using the transcriptome datasets of *G. hirsutum* (TM-1) [30], the expression profiles of 62 *GhPELs* with FPKM ≥1 in at least one of the 8 investigated tissues were shown in Fig. 5. The other 21 *GhPELs*, including all of the members (12) of the subfamily II, were very low or not expressed in all of the investigated tissues and developmental stages and 15 genes from gene duplication events, indicating that functional redundancy or pseudogenes existed in the *GhPEL* family.

Based on a clustering analysis, the 62 *GhPELs* were divided into 4 major patterns (A-D) (Fig. 5). Cluster A contained 29 genes, all belonging to subfamily IV and dominantly expressed in the flower, especially in the stamen, with very low expression in other tissues. In cluster B, all 4 *GhPELs* (*GhPEL28_At*, *GhPEL37_At*, *GhPEL63_Dt* and *GhPEL66_Dt*) from subfamily V were shown to have higher expression levels in the leaf than in other tissues. Cluster C contained 16 *GhPELs* from subfamily V and I general expression in all of the tissues, with some genes primarily expressed in fiber, stamen and other tissues. Cluster D was composed of another 13 *GhPELs*, from subfamily V and III, with lower expression in fiber than vegetative and reproductive organs. The gene expression pattern could manifest functions of genes, in part. The dominant expression of *GhPELs* in cluster A indicated that these genes performed crucial and conserved functions in the development of the flower, especially the stamen, which was concordant with *PLLs* generally expressed in flower and several *PLLs* highly expressed in pollen in Arabidopsis [19]. Some *GhPELs* in cluster C, including the *GhPEL* gene (*GhPEL48_Dt*) that was reported to regulate fiber elongation [16], showed higher expression in fiber than other tissues, which hinted that these *GhPELs* might regulate fiber development.

### Expression characterization of *GhPELs* in anther development

**Fig. 6:**
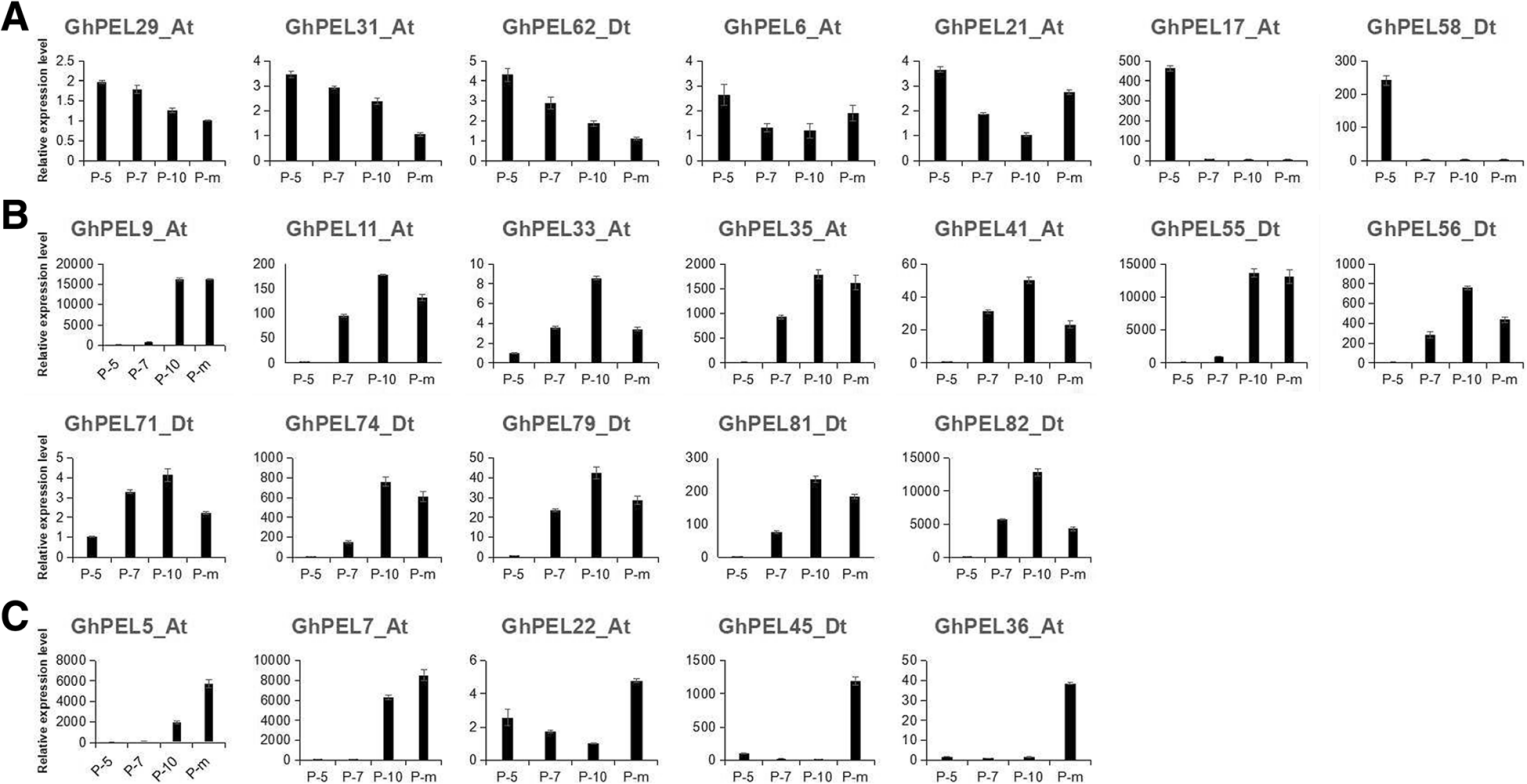
Expression patterns of *GhPELs* in different stages of anthers via qRT-PCR. P-5, P-7, P-10 and P-m represent anthers at the meiosis stage, mononucleate stage, binucleate stage and mature stage, respectively, in the cotton cultivar (CCRI040029). **a**-**c**: High expression in the meiosis stage, binucleate stage and mature stage of anthers. The error bars show the standard deviation of three biological replicates

To explore the expression features of *GhPELs* in anther development, using qRT-PCR, we identified the expression of 24 *GhPELs* in four stages of anther development, including meiosis stage, mononucleate stage, binucleate stage and mature stage (Fig. 6). The expression levels of 7 *GhPELs* (*GhPEL29_At*, *GhPEL31_At*, *GhPEL62_Dt*, *GhPEL6_At*, *GhPEL21_At*, *GhPEL17_At* and *GhPEL58_Dt*) were highest in the meiosis stage and decreased in subsequent stages, especially *GhPEL17_At* and *GhPEL58_Dt* with plummeting expression after the meiosis stage (Fig. 6a). Twelve *GhPELs* showed a constantly increasing expression to a peak at the binucleate stage, then decreased at the mature stage (Fig. 6b). The remaining 5 genes (*GhPEL5_At*, *GhPEL7*_*At*, *GhPEL22*_*At*, *GhPEL36*_*At* and *GhPEL45*_*Dt*) exhibited higher levels at the mature stage than at the other three stages, especially *GhPEL36*_*At*, which displayed a very low expression at the other three stages except for the mature stage (Fig. 6c). These results indicated that some *GhPELs* performed various and significant functions in the different development stages of anthers.

### Characterization of *GhPEL* gene expression in fiber development

**Fig. 7:**
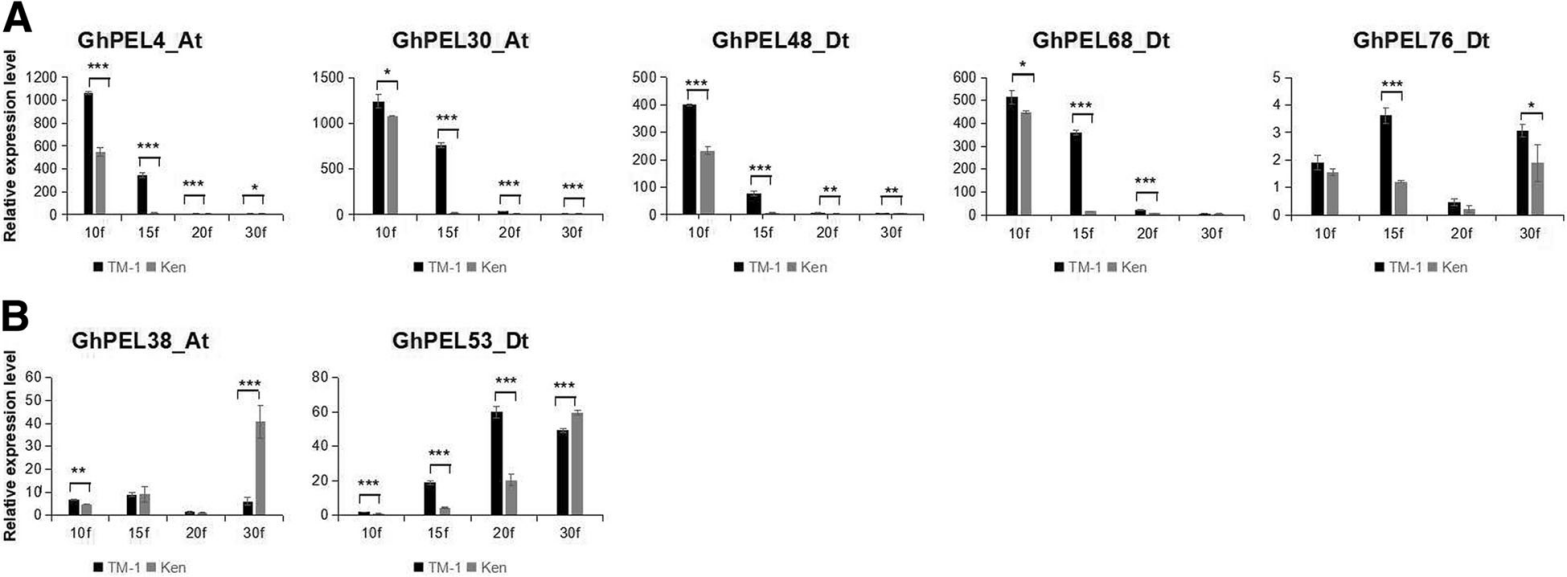
Expression analysis of *GhPELs* in fiber developmental stages between two cotton cultivars via qRT-PCR. TM-1 and Ken are the upland cotton cultivars TM-1 and kenN 27–3. 10f, 15f, 20f and 30f represent 10 DPA, 15 DPA, 20 DPA and 30 DPA fibers. **a**: Higher expression in TM1 than kenN 27–3 at different fiber elongation stages. **b**: Higher expression in kenN 27–3 than TM1 in 30 DPA fibers. Single, double and triple asterisks represent significant differences at the levels of 0.05, 0.01 and 0.001, respectively. The error bars show the standard deviation of three biological replicates

Two cultivars with significantly different fiber lengths (TM-1, with longer fibers, and kenN27–3, with shorter fibers) were chosen to explore the function of *GhPELs* in fiber development [34]. The expression of 7 genes (*GhPEL4_At*, *GhPEL48_Dt*, *GhPEL30_At*, *GhPEL68_Dt*, *GhPEL76_Dt*, *GhPEL38_At*, and *GhPEL53_Dt*), most of which exhibit higher expression in fiber than other organs, was analyzed in 10 DPA, 15 DPA, 20 DPA and 30 DPA fibers of TM-1 and kenN27–3. The results revealed that 5 genes (*GhPEL4_At*, *GhPEL48_Dt*, *GhPEL30_At*, *GhPEL68_Dt* and *GhPEL76_Dt*) displayed higher expression levels in TM-1 than in kenN27–3 in all stages and showed a significant difference in at least one stage, especially in the rapid elongation stage of fiber (10 DPA and 15 DPA) (Fig. 7a). *GhPEL38_At* and *GhPEL53_Dt* exhibited remarkably higher expression in fiber elongation stages and lower expression in the secondary wall thickening stage in TM-1 than kenN 27–3 (Fig. 7b). According to these results, these *GhPELs* might play an important role in fiber elongation.

### Responses of *GhPEL* genes to IAA treatment

**Fig. 8:**
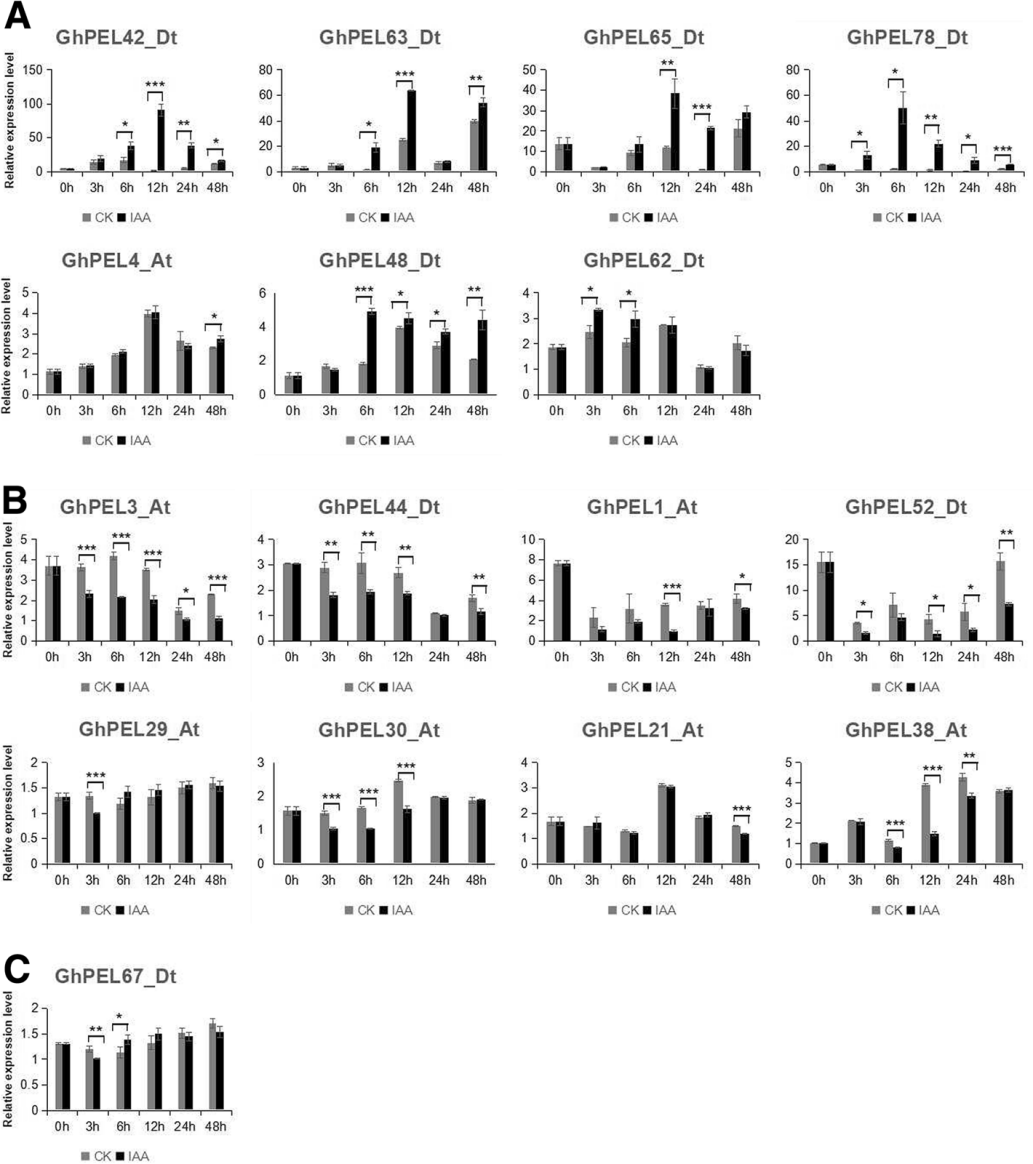
Expression analysis of *GhPELs* in the leaves under IAA treatment via qRT-PCR. 0 h, 3 h, 6 h, 12 h, 24 h and 48 h indicate hours after IAA treatment. **a**: Higher expression under IAA treatment than in CK at different IAA treatment times. **b**: Lower expression under IAA treatment than in CK at different IAA treatment times. **c**: No difference in expression between IAA treatment and CK at all IAA treatment times. Single, double and triple asterisks represent significant differences at the levels of 0.05, 0.01 and 0.001, respectively. The error bars show the standard deviation of three biological replicates

Studies have shown that auxin can regulate the activity of PEL [18, 19, 23]. Therefore, the expression features of 16 *GhPELs* were explored after IAA treatment (Fig. 8). Seven genes were notably up-regulated, and eight genes were notably down-regulated, mainly at 6 h and 12 h (Fig. 8a, b). The expression of *GhPEL67_Dt* showed notably earlier down-regulation and then up-regulation in response to IAA treatment at 3 h and 6 h, respectively (Fig. 8c). These results indicated that some *GhPELs* might take part in the biological pathways regulated by auxin.

## Discussion

### Characterization of *PEL* genes in cotton

Previous studies on *PEL* genes have mainly been conducted in plant pathogenic bacteria, focusing on pathogenic mechanisms [8, 35]. However, PEL-like genes have also been found in many plants, with genome-wide analyses revealing 26 *PEL* homologous genes in *Arabidopsis*, 12 in *Oryza sativa*, 46 in *Brassica rapa* and 30 in *Populus trichocarpa* [17, 19, 20, 36]. In the present study, a smaller number of *PEL* genes was identified in *Oryza sativa* (12 to 11), ignoring the Os06 g38520 genes due to exceeding the threshold (1e-10). We also identified 53 *PEL* homolog genes in *G. raimondii*, 42 in *G. arboreum* and 83 in *G. hirsutum*; these numbers are larger than those reported in most other species, indicating that gene expansion has occurred in the PEL gene family, especially in the IV and V subfamilies, during the evolution of cotton genomes. Gene duplication events have been reported to play an important role in gene expansion and both functional conservation and novelty in gene families [37, 38]. Our analysis of gene duplication in three cotton species showed that gene expansion in *G. hirsutum* and *G. arboreum* likely resulted primarily from segmental duplication (72.3% and 47.6%), whereas in *G. raimondii*, it resulted almost equally from tandem and segmental duplication (Additional file 5: Table S5). Genomic studies of cotton have revealed that two whole-genome duplication (WGD) events occurred in both *G. arboreum* and *G. raimondii*, one ancient and one recent event, at approximately 115–146 and 13–20 MYA, respectively [27–29]. Subsequently, polyploidization of A and D diploids and the emergence of *G. hirsutum* occurred, approximately 1–2 MYA [30]. In our study, analysis of the timing of the occurrence of segmental duplications showed that most of the events observed in *G. raimondii* and *G. arboreum* might have occurred from 8.48–145.46 MYA, except for GrPEL3/GrPEL13 (148.15 MYA) and GrPEL31/GrPEL43 (171.46 MYA), suggesting that these duplication events might have derived from the two WGD events. In *G. hirsutum*, the segmental duplication events most likely occurred before the hybridization of the two extant ancestors, according to the range of divergence times (1.00–32.75 MYA) and average time (10.24 MYA). The uneven chromosomal distribution of the *PEL* genes in the three cotton species might be due to the occurrence of these gene duplication events in the evolution of cotton.

Phylogenetic analysis indicated that the *PEL* genes could be classified into five subfamilies, with the largest members in subfamilies IV and V (Fig. 1, Additional file 2: Table S2). The *PELs* of *Oryza sativa* were located a long distance from those of the other plants, which might be related to different functions of these proteins between monocots and dicots [39]. All of the *GhPELs* exhibited the conserved Pec_lyase_C domain, with highly conserve Ca^2+^-binding, disulfide bonds, catalysis and substrate binding amino acid sites (Additional file 6: Figure S1 and Additional file 7: Fig. S2). The conserved motif analysis showed that the six motifs existed in most of the *GhPELs* (Fig. 2c). These data indicated that the *GhPELs* (especially the members of the same subfamily) might show a relatively conserved function in upland cotton growth. However, gene structure analysis revealed high diversity in the exon numbers of *GhPELs*, which could be related to diversification of their functions (Fig. 2b) [19, 40]. The similarities and differences of the gene structures, domains and motifs of *GhPELs* might be related to conservation and subfunctionalization, resulting from their long evolutionary history and gene duplication in cotton [37, 41].

### Role of *GhPELs* in plant growth and development

PELs are cell wall-modifying enzymes that can cleave α-1,4-glycosidic linkages of demethylated HG, the structural polysaccharide of the primary cell walls and middle lamella in higher plants, via a β-elimination mechanism [5]. Highly methyl-esterified HG is secreted into the extracellular matrix during cell division and is then de-esterified by PMEs. Demethylated HG is degraded by PELs and PGs more easily, promoting the loosening of the cell wall and regulating cell growth, cell division and organ morphogenesis in plant growth and development [2, 42, 43]. These findings indicate that pectinases, including PGs, PAEs, PMEs and PELs, regulate the cell wall composition and structure, playing important roles in different developmental processes of plants.

Comprehensive analyses of *PEL* genes have been reported in *Arabidopsis*, *Brassica rapa* and *Populu*s, with most of these genes being expressed in flowers [9, 10, 17, 19, 20, 36]. In the present study, a transcriptomic analysis of *GhPELs* revealed that 90% of the members in subfamily IV were preferentially expressed in the stamen, in agreement with the above reports (Fig. 5). To further explore the differences in the expression of *GhPELs* in anther development, we analyzed the expression of 24 *GhPELs* in four developmental stages of anthers, with 7, 12 and 5 *GhPELs* showing peak expression levels in meiosis, binucleate and mature stages, respectively (Fig. 6). These results indicated that many *GhPELs* performed synergistic and diverse functions in the process of anther development in upland cotton. Similar findings have been reported in *Brassica rapa*, in which *BrPLL1*, *BrPLL8–1* and *BrPLL11–3* were expressed only at the mature pollen stage, while *BrPLL6* showed specific expression at the meiosis and tetrad stages of anthers, and in tomato, in which *LAT56* and *LAT59* displayed maximal expression levels in the mature anthers [9, 36]. The *PELs* expressed during anther development might function to facilitate the degradation of the primary cell wall in pollen mother cells (PMC) during the meiosis stage and take part in anther dehiscence, cell wall loosening in pollen, pollen tube elongation and the promotion of pollen penetration through style tissue degradation in mature anthers [11, 44–46].

Cotton fiber, which is a widely used natural fiber in the textile industry, is produced from a single differentiated epidermal cell of the ovule [47, 48]. In cotton fiber, pectin was produced beginning at anthesis, continuously through 19–20 DPA, and was only found only in the primary cell wall [49]. Here, we explored the expression features of 7 *GhPELs*, including 3 paralogous gene pairs with high expression in fiber, in two upland cotton cultivars showing significantly different fiber lengths. All 7 *GhPELs* exhibited significantly higher levels of expression in TM1 than kenN 27–3 during the rapid elongation stage of fiber (Fig. 7). However, *GhPEL38_At* and *GhPEL53_Dt* showed lower expression at 30 DPA, which was the secondary wall thickening stage. *GhPEL* (*GhPEL48_Dt*) has been shown to play an important role in fiber cell elongation through the degradation of pectin in the primary cell wall, facilitating cell wall loosening [16]. *GhPEL4_At*, *GhPEL30_At*, *GhPEL68_Dt* and *GhPEL76_Dt* appear to exhibit the same expression characteristics as *GhPEL48_Dt*, indicating that these genes might perform similar functions in fiber elongation. *GhPEL38_At* and *GhPEL53_Dt* showed expression levels at the secondary wall thickening stage that were different from those in the other 5 *GhPELs*, which is probably related to their different roles in secondary wall thickening in fiber. A study of *PtPLs* revealed that a *PtPL1–18* overexpression line exhibited much thinner secondary walls than control plants, which indicated that *PELs* could regulate the structure of secondary walls as well as primary cell walls [17]. All of these results indicated that these genes played a substantial role in fiber elongation and influence the quality of fiber in upland cotton.

Auxin is involved in acid-mediated changes in the cell wall, inducing the expansins and pectinases, leading to cell wall loosening and organ initiation [21, 50]. Previous reports have revealed that *PEL* genes take part in pathway responses to auxin to regulate cell elongation and differentiation and other plant developmental processes [18, 19, 24]. According to our analysis of cis-elements, all of the *GhPELs* contained at least one of the six auxin-responsive cis-elements (Additional file 8: Table S6). Therefore, we randomly chose 16 *GhPELs* to investigate the response to IAA treatment. All of these genes showed a response to IAA treatment, primarily at 6 h and 12 h (Fig. 8). Among these genes, *GhPEL30_At*, *GhPEL38_At* and *GhPEL48_Dt* also participate in the development of fiber, and *GhPEL62_Dt* is involved in anther development, especially at the meiosis stage. Previous studies have shown that auxin regulates the development of fiber elongation and the anther at early and late stages [51–53]. Our results revealed that *GhPELs* likely regulate plant growth and development by responding to the IAA signal.

Previous studies have shown that *PEL* genes play significant roles in different plant development processes, mainly by changing the cell wall composition and structure [1, 5]. PEL and pectin methylesterases can alter the cell wall composition, cell wall structure and cell wall loosening by regulating the content and status of methyl-esterified and de-esterified HG [54], thereby affecting plant morphology [1] fruit firmness, pathogen resistance [14], anther development [36] and fiber elongation [16]. Moreover, auxin participates in molding the cell wall and could regulate PEL enzyme activity [24]. According to our gene expression analysis, *GhPELs* showed diverse gene expression features in different tissues. In particular, we analyzed the expression of *GhPELs* in anther and fiber development and in response to IAA treatment and inferred that they played roles in these processes likely by altering cell wall composition, cell wall structure and cell wall loosening. However, the regulatory networks and functions of the *GhPELs* require further studies.

## Conclusions

We performed a genome-wide analysis of the *PEL* gene family in *G. raimondii*, *G. arboreum*, and *G. hirsutum*. According to analyses of phylogeny, chromosomal location and gene duplication events, *PEL* genes were divided into 5 subfamilies, and it could be inferred that *PEL* gene expansion occurred due to gene duplication. All of the *GhPELs* exhibited the conserved Pec_lyase_C domain, and diverse gene structures were observed among them. The analysis of expression revealed that the *GhPELs* showed different expression features in different organs and developmental stages and could respond to IAA treatment. These results indicated that *PELs* played important roles in plant growth and development, especially in anther and fiber development and the auxin signaling pathway. The results of our study provide a fundamental basis for further research on the functions of *PEL* genes in cotton.

## Methods

### Identification of *PELs* in cotton

The conserved Pec_lyase_C (Pfam00544) domain was downloaded from Pfam (http://pfam.xfam. org) [55] and used to search against predicted proteins of *G. raimondii* (JGI_v2.1), *G. arboreum* (BGI_v2.0) and *G. hirsutum* acc. TM-1 (NAUNBI_v1.1) from the CottonGen website (https://www.cottongen.org/icgi/home), using HMMER 3.0 [56]. We also searched the Arabidopsis genome (TAIR 10, http://www.arabidopsis.org) using the Pec_lyase_C domain. The E-value threshold for the HMMER search was set at 1e − 10 to obtain possible PEL proteins. Then, the normal mode of the SMART database (http://smart.embl-heidelberg.de/) was used to confirm every putative GhPEL protein with a Pec_lyase_C domain [57]. Only the sequences containing a conserved Pec_lyase_C domain were employed for further analysis.

The theoretical molecular weight (Mw), isoelectric point (pI), grand average of hydropathicity (GRAVY) and subcellular localization of the predicted GhPELs were predicted using ExPASy (http://cn.expasy.org/tools) and the CELLO v2.5 server (http://cello.life. nctu.edu.tw/) [58, 59].

### Sequence alignment and phylogenetic analysis

Multiple alignment of all the predicted PEL protein sequences from the three *Gossypium* species, *Arabidopsis*, *Corchorus olitorius*, *Theobroma cacao*, *Oryza sativa* and *Populus* genomes was performed using ClustalX 2.0 [60]. An unrooted phylogenetic tree was generated using the neighbor-joining (NJ) method and the amino acid p-distance model in MEGA 6.0 [61]. Bootstrap resamplings (1000) were used to assess the reliability of interior branches.

### *PEL* gene locations on cotton chromosomes and gene duplication analysis

The physical chromosome locations of all *PEL* genes were obtained from the genome sequence databases of the three *Gossypium* species and visualized with MapInspect (http://www.plantbreeding.wur.nl/uk/ software-mapinspect.html). The predicted PEL proteins of the three cotton species were aligned with ClustalW2 at EMBL-EBI (http://www.ebi.ac.uk/Tools/msa/clustalw2/). Gene duplication was confirmed if the following conditions were satisfied: (1) the coverage of the alignment was > 80% of the longer gene; (2) the identity of the aligned regions was > 80%; and (3) genes separated by five or fewer gene loci with a distance of less than 100 kb on the same chromosome were considered to represent a tandem duplication [62–64]. A diagram of segmental duplication was drawn with Circos 0.69 [65]. Nonsynonymous (Ka) and synonymous substitution (Ks) rates were calculated using DnaSp V5.0 software, employing the full-length gene sequences of the segmental duplicated *PEL* gene pairs from the three cotton species aligned by ClustalX 2.0 [66]. The Ka/Ks ratio was assessed to determine the molecular evolutionary rates of each gene pair. In general, Ka/Ks < 1 indicates purifying selection; Ka/Ks = 1 indicates neutral selection; and Ka/Ks > 1 indicates positive selection. The divergence times of these gene pairs were estimated using the formula “t = Ks/2r”, with r (2.6 × 10^− 9^) representing neutral substitution [30, 67].

### Multiple sequence alignments and conserved Pec_lyase_C domain analysis

The GhPEL protein sequences were employed for multiple sequence alignments with ClustalX 2.0. SMART was applied to determine the conserved Pec_lyase_C domains. The SignalP 4.0 server (http://www.cbs.dtu.dk/services/SignalP/) was used to predict potential signal peptides within the GhPEL proteins (cutoff > 0.45) [68].

### Gene structure and conserved motif analysis

The genomic sequences and positions of the exons and introns of *GhPELs* were employed to visualize PEL exon-intron structures on the Gene Structure Display Server (GSDS) (http://gsds.cbi.pku.edu.cn/) [69]. The MEME program was used to analyze the conserved motifs of the GhPEL protein sequences with the following parameters: site distribution, zero or one occurrence per sequence; number of motifs, 6; and motif width, 6–50 [70].

### Analysis of cis-elements of upstream sequences

To determine the cis-elements of the predicted promoters, the 2000 bp genomic DNA sequences upstream of the initiation codon (ATG) of all *GhPELs* were employed to search the PLACE database (http://www.dna.affrc.go.jp/PLACE/signalscan.html).

### Gene expression pattern analysis

The expression levels of *GhPELs* in different tissues were obtained from previously reported transcriptome data [30]. The *GhPELs* with an FPKM > 1 were used for further expression analysis [71–73]. The expression clusters were calculated using Mev4.6.2 software (http://www.tm4.org/mev.html).

### Plant materials and treatments

Three *G. hirsutum* cultivars (TM-1, kenN 27–3 and CCRI040029) were field grown in Anyang, Henan province, China. The fibers were separated from the ovules at 10, 15, 20 and 30 DPA. The anthers of CCRI040029 were harvested when the flower buds had grown to 5 mm, 7 mm and 10 mm and at anthesis [74].

TM-1 was also grown in a climate-controlled greenhouse (light/dark cycle: 16 h at 28 °C/8 h at 22 °C) and was employed to investigate the responses to IAA treatment. Seedlings exhibiting third true leaves were sprayed with 100 mM IAA and water as a control group. The leaves of ten seedlings in each group were collected at 0 h, 3 h, 6 h, 12 h, 24 h and 48 h after treatment. All samples were immediately frozen in liquid nitrogen and stored at − 80 °C.

### RNA extraction and qRT-PCR analysis

Total RNA of collected samples was extracted using the Tiangen RNAprep Pure Plant kit (Tiangen, China) according to the manufacturer’s instructions. First-strand cDNA was synthesized via reverse transcription of 1 μg of total RNA using the PrimeScript RT Reagent kit (Takara, Japan). Primer 5.0 software was used to design the gene-specific primers for qRT-PCR (Additional file 9: Table S7). The histone-3 gene (AF024716) was employed as an internal reference control. The qRT-PCR experiments were performed using SYBR Premix Ex Taq (Takara) on an ABI 7500 real-time PCR system (Applied Biosystems, USA) with three replicates. The details of the protocol were as follows: (Step 1) initial denaturation step of 30 s at 95 °C, (Step 2) 40 cycles of 5 s at 95 °C, 34 s at 60 °C and (Step 3) melting curve analysis. The 2^-△△CT^ method was used to calculated the relative expression levels of *GhPELs* [75]. T-tests were employed for statistical analyses.

## Additional files

Additional file 1: **Table S1.** Detailed parameters of the PEL proteins from three cotton species. (XLS 21 kb)

Additional file 2: **Table S2.** Numbers of PELs in the five subfamilies in different species. (XLS 9 kb)

Additional file 3: **Table S3.** Chromosome locations of *PEL* genes in three cotton species. (XLS 50 kb)

Additional file 4: **Table S4.** Gene duplication events of *PEL* genes in three cotton species. (XLS 11 kb)

Additional file 5: **Table S5.** Ka/Ks analysis and divergence times of segmentally duplicated *PEL* gene pairs of three cotton species. (XLS 31 kb)

Additional file 6: **Figure S1.** The conserved Pec_lyase_C domain and signal peptide of GhPEL proteins. Left: Phylogenetic analysis of GhPEL proteins using MEGA 6.0 via the neighbor-joining (NJ) method with 1,000 bootstrap replicates. Right: Conserved domains of GhPEL proteins. Light-blue filled boxes represent the Pec_lyase_C domain, and green filled boxes represent the signal peptide. (TIF 1224 kb)

Additional file 7: **Figure S2.** Conserved amino acid sites of the GhPEL proteins. Multiple alignment analysis of GhPEL proteins. The pink, blue, green and red boxes and asterisks represent the substrate binding sites, Ca^2+^-binding sites, catalysis site and disulfide bond site, respectively. (TIF 6096 kb)

Additional file 8: **Table S6.** Cis-elements related to auxin in the promoters of *GhPEL* genes. (XLS 50 kb)

Additional file 9: **Table S7.** Primer pairs used in qRT-PCR analysis. (XLS 25 kb)

## Abbreviations

AA: Amino acid
DPA: Days postanthesis.
FPKM: Fragments per kilobase of transcript per million fragments
*Ga*: *Gossypium arboreum*
*Gh*: *Gossypium hirsutum*
*Gr*: *Gossypium raimondii*
Ka: Nonsynonymous substitution rate
Ks: Synonymous substitution rate
MYA: Million years ago
PEL/PLL: Pectate lyase
PMC: Pollen mother cells
qRT-PCR: Quantitative real-time polymerase chain reaction
WGD: Whole-genome duplication
