# Supplementary figures and images for: Genome-wide identification and expression analyses of the pectate lyase (PEL) gene family in cotton (Gossypium hirsutum L.)
Source: BMC Genomics. 2018 Sep 10;19:661. doi: 10.1186/s12864-018-5047-5 (PMC6131898; doi:10.1186/s12864-018-5047-5)

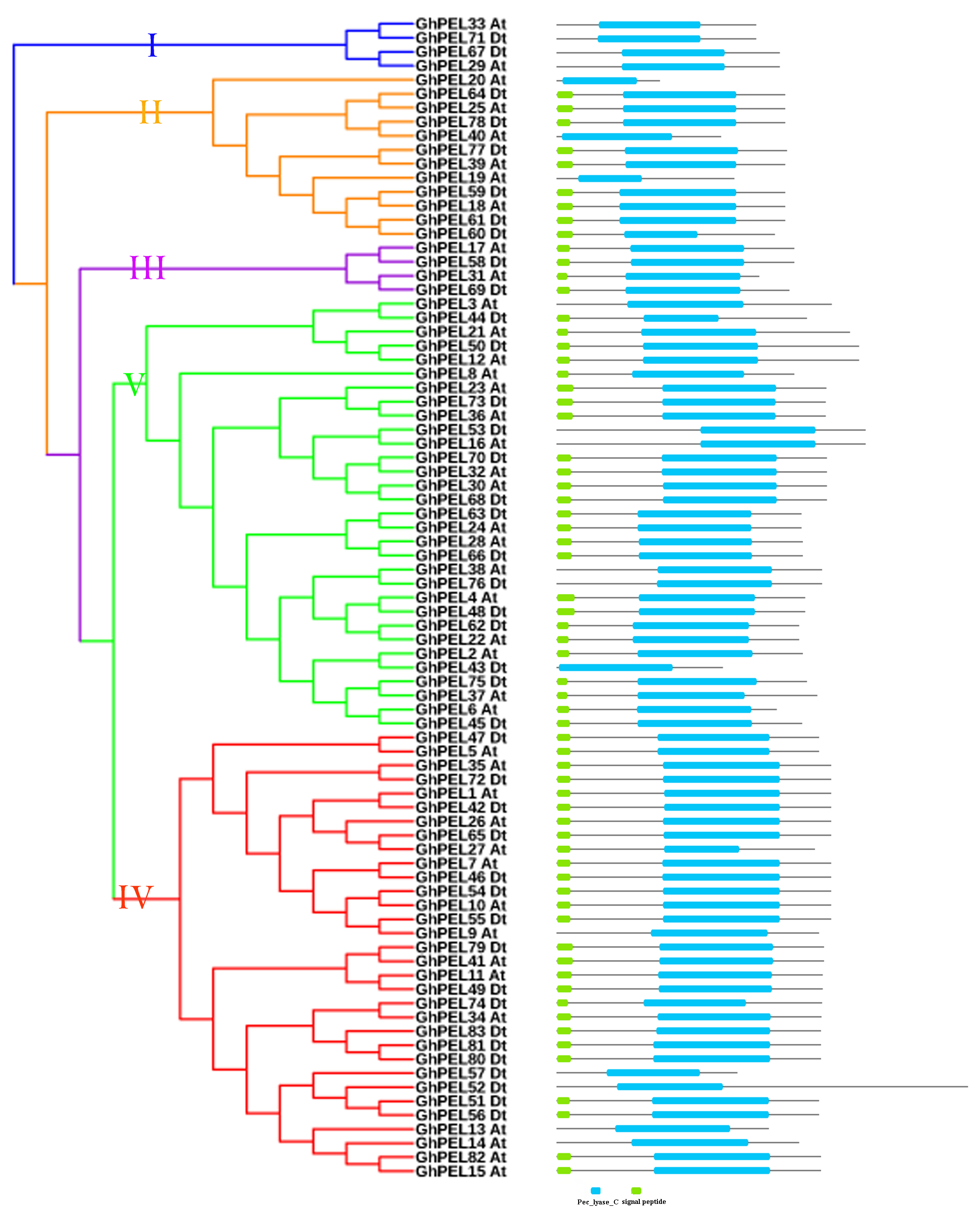

Supplement: Supplementary file 6 — Figure S1. The conserved Pec_lyase_C domain and signal peptide of GhPEL proteins. Left: Phylogenetic analysis of GhPEL proteins using MEGA 6.0 via the neighbor-joining (NJ) method with 1,000 bootstrap replicates. Right: Conserved domains of GhPEL proteins. Light-blue filled boxes represent the Pec_lyase_C domain, and green filled boxes represent the signal peptide. (TIF 1224 kb) [file 12864_2018_5047_MOESM6_ESM.tif]

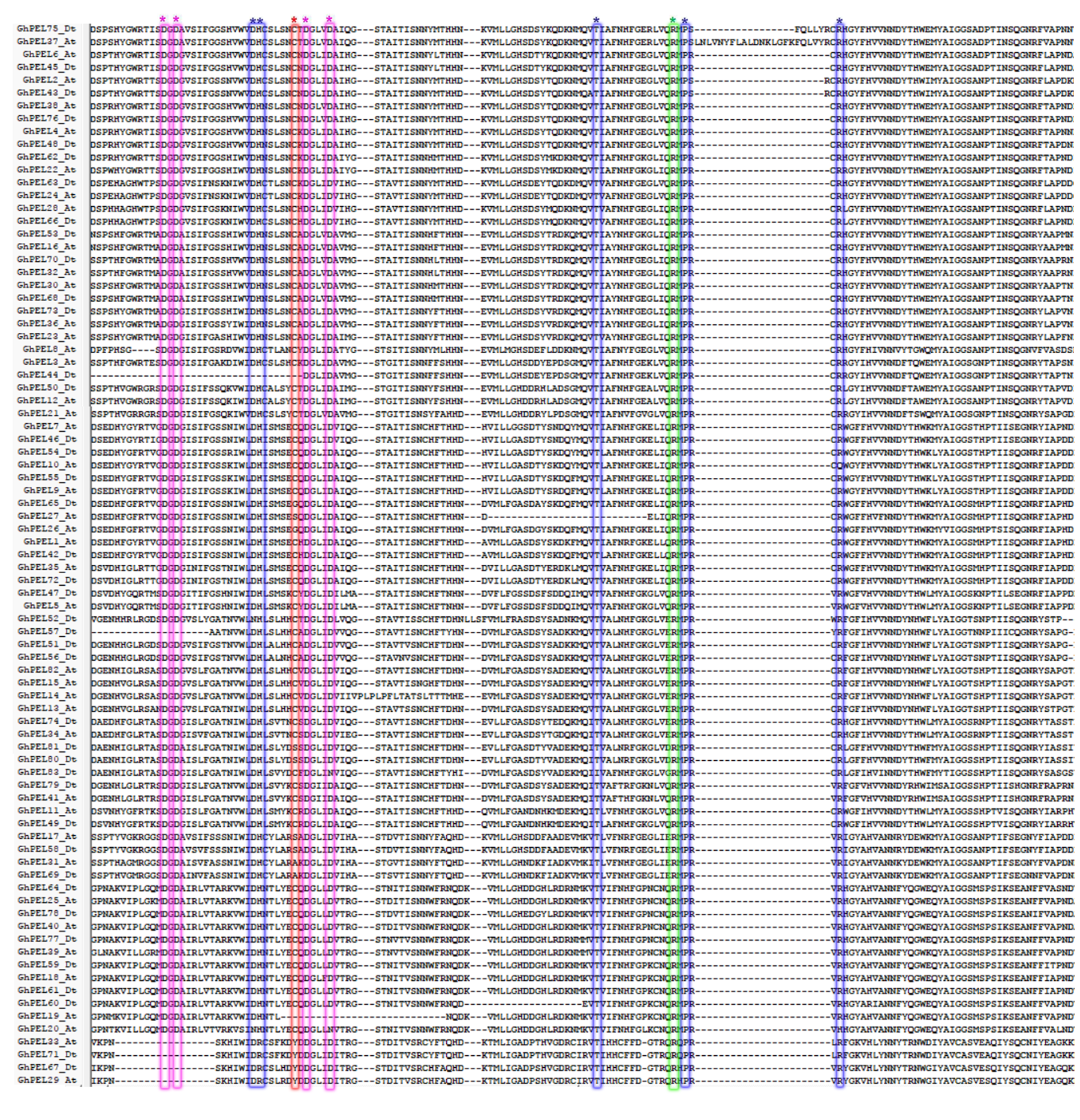

Supplement: Supplementary file 7 — Figure S2. Conserved amino acid sites of the GhPEL proteins. Multiple alignment analysis of GhPEL proteins. The pink, blue, green and red boxes and asterisks represent the substrate binding sites, Ca2+-binding sites, catalysis site and disulfide bond site, respectively. (TIF 6096 kb) [file 12864_2018_5047_MOESM7_ESM.tif]
